# Acute Pneumonitis Associated With the Inhalation of Pyrethroid-Based Domestic Insecticides

**DOI:** 10.7759/cureus.43200

**Published:** 2023-08-09

**Authors:** Toyoshi Yanagihara, Takuya Nakagawa, Takehito Fukushima, Yuki Moriuchi, Hiroaki Ogata, Akiko Ishimatsu, Junji Otsuka, Masako Kadowaki, Atushi Moriwaki, Makoto Yoshida

**Affiliations:** 1 Department of Respiratory Medicine, National Hospital Organization Fukuoka National Hospital, Fukuoka, JPN; 2 Department of Allergology, National Hospital Organization Fukuoka National Hospital, Fukuoka, JPN; 3 Department of Internal Medicine, National Hospital Organization Fukuoka National Hospital, Fukuoka, JPN

**Keywords:** domestic insecticide, acute respiratory failure, inhaled pyrethroid, pulmonary toxicity, acute pneumonitis

## Abstract

We report a 72-year-old female who presented to our hospital with a worsening cough and dyspnea that had emerged a week earlier following the accidental inhalation of a significant quantity of spray-type imiprothrin (a synthetic pyrethroid)-based insecticide in her bathroom. She exhibited acute respiratory failure necessitating 4 L/minute of nasal oxygen at maximum. Chest CT images showed diffuse centrilobular ground-glass opacities with mosaic attenuation and consolidation areas in the lower lobes of both lungs. The patient was diagnosed with acute pneumonitis due to insecticide inhalation, and her symptoms improved following methylprednisolone pulse and alpha-tocopherol therapy. Generally, the accidental inhalation of aerosolized pyrethroids does not induce significant respiratory symptoms, and case reports on pulmonary toxicity related to pyrethroid inhalation are scarce. This case report underscores the need to include inhaled pyrethroid insecticides in the differential diagnosis of patients with acute pneumonitis and suggests that methylprednisolone and alpha-tocopherol therapy can be beneficial for treating this condition.

## Introduction

Pyrethroids, the synthetic equivalents of pyrethrin, a natural pesticide derived from *Chrysanthemum cinerariaefolium* flowers, constitute a vital class of insecticides [1]. These substances permeate various consumer products, encompassing home and garden insecticides, sprays, shampoos for pets, lice treatments, and mosquito repellents. Pyrethroid insecticides have become popular because of their effectiveness against insects and their low toxic effects in humans. Despite this, there have been several reports of toxicity to humans in both occupational exposure and deliberate ingestion poisoning. Commonly reported symptoms include facial paresthesia, skin itching, skin burning, dizziness, nausea, vomiting, and more severe cases of muscle fasciculations [2]. On the other hand, there are few reports regarding pulmonary toxicity related to pyrethroids. Here, we report a case of acute pneumonitis associated with the inhalation of pyrethroid-based domestic insecticides.

## Case presentation

A 72-year-old female presented to our hospital due to worsening symptoms of cough and dyspnea. These symptoms emerged one week prior, following the accidental inhalation of a substantial quantity of spray-type imiprothrin (synthetic pyrethroid)-based insecticide named "Goki Jet Pro®" in her bathroom. She had deployed the spray insecticide for over 30 seconds in the bathroom enclosed without adequate ventilation, which resulted in eye and throat irritation. Her condition worsened despite azithromycin prescription from a local practitioner three days prior. The patient's medical history included managed hypertension, hypothyroidism, and osteoporosis.

**Figure 1:**
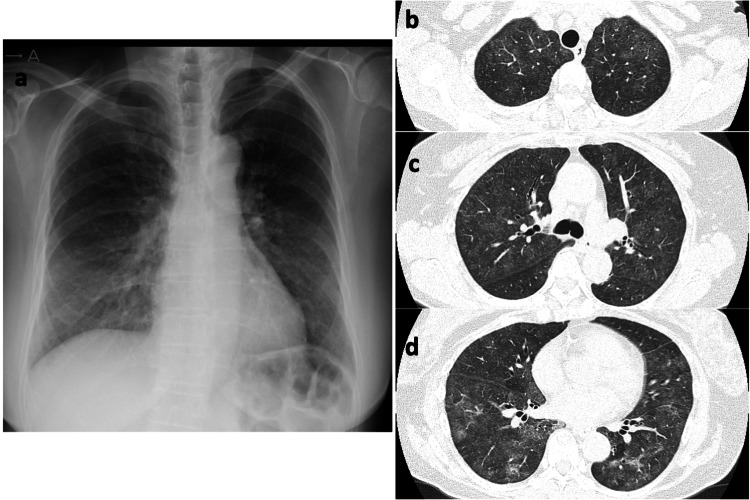
Chest X-ray and chest CT images of the patient. (a) Ground-glass opacities in bilateral lower lung fields on chest X-ray and (b-d) diffuse centrilobular ground-glass opacities with mosaic attenuation, along with areas of consolidation in the lower lobes of both lungs on chest CT

On presentation, she exhibited tachypnea with a rate of 30 breaths/minute, oxygen saturation (SpO_2_) of 89% on room air, body temperature of 36.1°C, heart rate of 90 beats/minute, and blood pressure of 155/83 mmHg. Physical examination identified late inspiratory crackles in bilateral lower dorsal areas. Arterial blood gas showed partial pressure of oxygen (PO_2_) of 50.8 mmHg, partial pressure of carbon dioxide (PCO_2_) of 34.8 mmHg, bicarbonate (HCO_3_-) of 25.1 mmol/L, and pH of 7.48. Laboratory investigations included a WBC count of 10.03×10^3^/μL, hemoglobin (Hb) of 12.4 g/dL, platelet (PLT) of 307×10^3^/μL, C-reactive protein (CRP) of 9.76 mg/dL, lactate dehydrogenase (LDH) of 264 U/L, blood urea nitrogen (BUN) of 11.2 mg/dL, creatinine (Cr) of 0.70 mg/dL, and Krebs von den Lungen-6 (KL-6) of 490 U/mL. Imaging demonstrated ground-glass opacities in bilateral lower lung fields on chest X-ray and diffuse centrilobular ground-glass opacities with mosaic attenuation, along with areas of consolidation in the lower lobes of both lungs on chest CT (Figure 1). The FilmArray respiratory panel was negative for all pathogens, including SARS-CoV-2. The patient was admitted with respiratory failure necessitating 2 L/minute of nasal oxygen, which increased to 4 L/minute at night.

**Figure 2:**
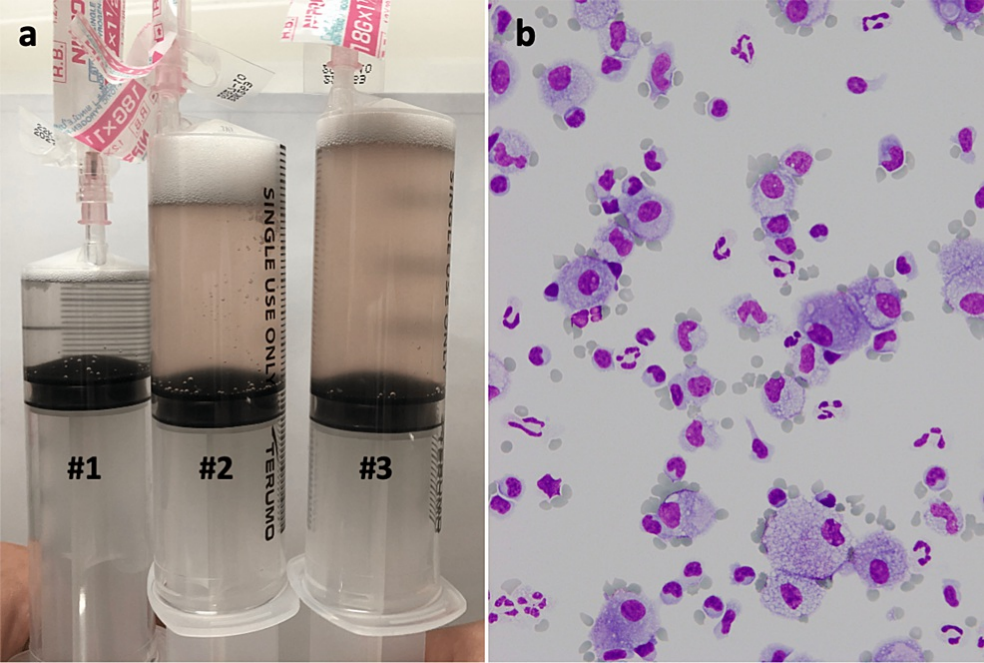
Bronchoalveolar lavage fluid of the patient. (a) Bronchoalveolar lavage (BAL) from the right B4 retrieved 77/150 mL, which was pale red during the second and third collections, suggesting mild alveolar hemorrhage. (b) Diff-Quik staining of BAL cells showing increased lymphocytes (38.4%) and neutrophils (33.3%) with red blood cells

Given her clinical manifestation, the preliminary diagnosis was acute pneumonitis due to insecticide inhalation. Bronchoscopy was performed the following day. Bronchoalveolar lavage (BAL) from the right B4 retrieved 77/150 mL, which was pale red during the second and third collections, suggesting mild alveolar hemorrhage (Figure 2). Bronchoalveolar lavage fluid (BALF) analysis showed an increased total cell count (4.4×10^5^/mL), with 27.9% macrophages, 38.4% lymphocytes, 33.3% neutrophils, and 0.4% eosinophils. The cluster of differentiation (CD) 4/CD8 ratio was 3.87. The BALF did not show any bacteria or acid-fast bacilli.

**Figure 3:**
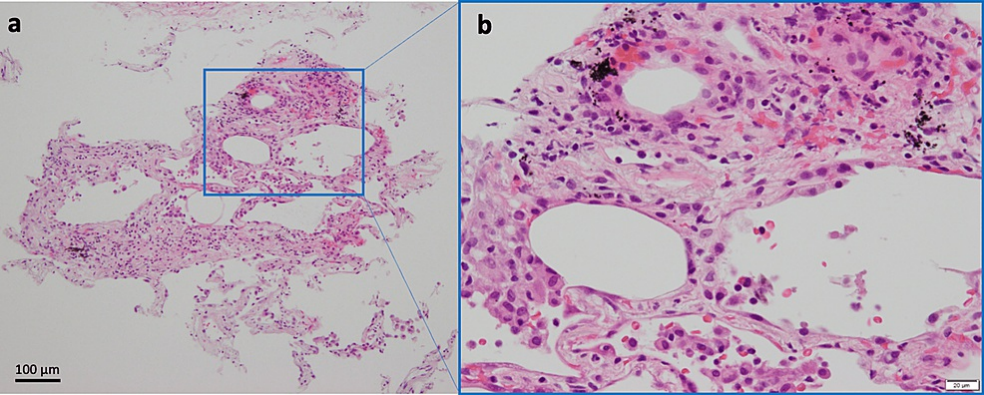
Histological examination of transbronchial lung biopsy. (a) Hematoxylin and eosin staining of transbronchial lung biopsy from the right B2b and B8b. The alveolar septa exhibit edema and infiltration of inflammatory cells, but no fibrosis is observed. No obvious evidence of lipid/cholesterol crystal-laden macrophages or granulomas. (b) Higher magnification

Transbronchial lung biopsy was performed from the right B2b and B8b. Histopathology indicated edema and inflammatory cell infiltration in the alveolar septa, although no fibrosis was observed (Figure 3). Lipid or cholesterol crystal-filled macrophages or granulomas were not observed.

**Figure 4:**
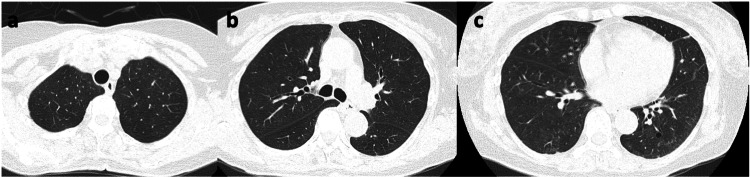
Chest CT images of the patient before discharge. (a-c) Chest CT scans before discharge revealed improved ground-glass opacities and consolidation observed on admission

Subsequently, the patient was initiated with intravenous methylprednisolone 500 mg as steroid pulse therapy for three days immediately post bronchoscopy. On the fourth day of admission, alpha-tocopherol 600 mg/day was added due to persistent respiratory failure. On the fifth day, the methylprednisolone dose was reduced to 62.5 mg. The patient recovered from respiratory failure on day 9, leading to a further reduction in methylprednisolone to 8 mg. Subsequent CT images on day 10 showed improved ground-glass opacity/infiltration (Figure 4), resulting in the discontinuation of methylprednisolone. The patient was discharged on day 12.

## Discussion

Imiprothrin, a synthetic pyrethroid first identified by Itaya, demonstrates superior knockdown efficacy against cockroaches compared to other synthetic pyrethroids. The safety data sheet for imiprothrin indicates an inhalation lethal dose of 50 (LC50) exceeding 1.2 mg/L in rats, and it is classified under toxicity category III, denoting slight toxicity and irritation [3]. This suggests that imiprothrin is generally deemed safe for human use under normal conditions. Nonetheless, in instances of excessive utilization, such as the case, the concentration may escalate to potentially toxic levels, as the patient had felt irritation in her eyes and throat.

Two potential pathomechanisms underpin the pulmonary toxicity of pyrethroids in humans. Firstly, pyrethroids predominantly function by blocking voltage-gated sodium channels in insects [4]. Several mammalian voltage-gated sodium channel isoforms are similarly sensitive to pyrethroid exposure, albeit with reduced sensitivity [5]. Both alveolar epithelial cell types I and II possess sodium inward transport channels [6]. Pyrethroids can affect sodium channels on these cells and disrupt osmotic gradients, potentially causing bronchial/epithelial mucosal edema [7]. ﻿Secondly, pyrethroids undergo metabolism by cytochrome P450 enzymes [8]. At high concentrations, pyrethroids can accumulate, exceeding the metabolic capacity of these enzymes, thereby inducing oxidative stress and leading to cytotoxicity [9]. Animal studies demonstrated that high-dose pyrethroids induce pulmonary edema, interstitial inflammation, and lymphocyte infiltration in the lung [10]. Given the cytotoxicity caused by oxidative stress, anti-oxidants have therapeutic potential. In an animal study, N-acetyl cysteine has a beneficial effect on alpha-cypermethrin-induced pulmonary toxicity [9]. The protective properties of ascorbic acid (vitamin C) against liver, kidney, brain, and heart tissue damage and alpha-tocopherol (vitamin E) against kidney and lung tissue damage have been reported in rodent studies [10]. Despite the absence of reports regarding the use of these anti-oxidants for pulmonary toxicity in humans, we chose to administer alpha-tocopherol to the patient based on its safety profile.

Methylprednisolone pulse therapy was administered to the patient. Vorselaars et al. reported three cases of severe pulmonary toxicity post inhalation of pyrethroid-based insecticides, requiring hospitalization, one of which was fatal [11]. Chest CT images from two cases suggested a nonspecific interstitial pneumonitis (NSIP) pattern, while the fatal case indicated a diffuse alveolar damage (DAD) pattern [11]. All three patients were treated with corticosteroids, and two showed substantial improvement. In a pediatric case of lung injury caused by the accidental ingestion of meperfluthrin, a type of pyrethroid, inhaled corticosteroids were employed [12]. If lung injury is mild, inhaled corticosteroids could be a suitable alternative, given their lesser side effects compared to systemic corticosteroid treatment. However, the use of corticosteroids in treating acute pneumonitis resulting from chemical agent inhalation is controversial [13]. While systemic corticosteroids appear beneficial in the present case, more evidence is needed.

Previous literature points toward the possibility of hypersensitivity pneumonitis arising from pyrethroid exposure, particularly among pet groomers frequently utilizing pyrethroid-based pet sprays [14,15]. In the current case, considering the patient's clinical progression post admission and based on both BALF cytology and histological analysis, acute pneumonitis attributable to inhaled insecticides was clinically diagnosed. However, the CT images still fit a typical pattern of nonfibrotic hypersensitivity pneumonitis [16]; the case requires close follow-up. A multidisciplinary discussion (MDD) could have been conducted to rule in or out the diagnosis of nonfibrotic hypersensitivity pneumonitis.

## Conclusions

In conclusion, this case study highlights the potential for severe pulmonary toxicity following the inhalation of pyrethroid-based insecticides, an often-overlooked hazard in nonoccupational environments. Our findings underscore the need for increased awareness among healthcare providers regarding this possibility in the differential diagnosis of acute pneumonitis. Additionally, the successful use of methylprednisolone and alpha-tocopherol therapy in managing this patient's condition provides a potential therapeutic avenue worth further exploration.
